# Spatial propagation of movement-related basal ganglia activity predicts parkinsonian motor state

**DOI:** 10.1093/brain/awag019

**Published:** 2026-01-20

**Authors:** Alberto Averna, Mario Sousa, Elena Bernasconi, Eduardo Martin Moraud, Claudio Pollo, Paul Krack, Hagai Bergman, Benoit Duchet, Gerd Tinkhauser

**Affiliations:** Department of Neurology, Bern University Hospital and University of Bern, Bern CH-3010, Switzerland; Department of Neurology, Bern University Hospital and University of Bern, Bern CH-3010, Switzerland; Department of Neurology, Bern University Hospital and University of Bern, Bern CH-3010, Switzerland; Department of Clinical Neurosciences, Lausanne University Hospital (CHUV) and University of Lausanne (UNIL), Lausanne CH-1011, Switzerland; NeuroRestore, Defitech Centre for Interventional Neurotherapies, CHUV, UNIL, and Ecole Polytechnique Fédérale de Lausanne (EPFL), Lausanne CH-1011, Switzerland; Department of Neurosurgery, Bern University Hospital and University of Bern, Bern CH-3010, Switzerland; Department of Neurology, Bern University Hospital and University of Bern, Bern CH-3010, Switzerland; The Hebrew University of Jerusalem, Edmond and Lily Safra Center for Brain Sciences, Jerusalem 9190401, Israel; Nuffield Department of Clinical Neuroscience and MRC Brain Network Dynamics Unit, University of Oxford, Oxford OX1 3TH, UK; Department of Neurology, Bern University Hospital and University of Bern, Bern CH-3010, Switzerland

**Keywords:** motor performance, Parkinson’s disease, movement-related synchronization, basal ganglia, local field potentials, deep brain stimulation

## Abstract

Movement-related gamma activity (> 60 Hz) in cortico-basal ganglia networks reflects pro-kinetic synchronization dynamics. While in the cortex these temporal dynamics are known to unfold spatially across topographically distributed networks, it remains unclear whether a similar spatial propagation occurs within the basal ganglia, and how such spatial encoding may contribute to both physiological and disease-related mechanisms. The subthalamic nucleus (STN) is a key integrative hub for motor processing within the basal ganglia-cortical circuitry. At rest, STN activity is topographically distributed according to its spectral frequency components.

To assess whether this spectral topography is dynamic and underlies movement encoding, we dissected the spatiotemporal properties of STN local field potentials recorded intraoperatively at rest and during movement across 63 hemispheres from patients with Parkinson’s disease. Using multi-contact deep brain stimulation leads, we captured high-resolution anatomical signal dynamics and contrasted a broad frequency spectrum (60–400 Hz), including high-gamma, fast-gamma, slow high-frequency oscillations and fast high-frequency oscillations. Moreover, we compared these signals to upper limb muscle activity and movement-related beta desynchronization, and examined their association with clinical impairment and levodopa responsiveness.

All sub-bands exhibited significant movement-related synchronization in both the contralateral and ipsilateral STN, however, with distinct magnitude and temporal dynamics. The presence and degree of temporal locking to muscle activity and inverse relationship to movement-related beta desynchronization also varied by sub-band. Importantly, each sub-band exhibited spatially segregated hotspots located within the STN that propagate primarily along the inferior-superior axis, yet in band-specific directions. This spatial propagation evolved throughout the movement period but temporally decoupled from synchronization magnitude, indicating that spatial dynamics reflect a distinct property relevant for motor encoding. Notably, propagation of frequencies above 110 Hz inversely correlated with dopamine-related motor improvement, suggesting that exaggerated spatial dynamics may reflect compensatory mechanisms secondary to neurodegeneration.

These findings demonstrated that synchronization within the basal ganglia is not a spatially static phenomenon but rather unfolds in space which expands on the current understanding of the basal ganglia mechanism. Propagation of movement-related activity may serve as a potential marker for motor impairment in Parkinson’s disease, opening new avenues for spectro-behavioural research and spatially informed neuromodulation strategies.

## Teaser

Averna *et al.* show that movement-related activity in the STN of patients with Parkinson’s disease propagates spatially during motion, with the degree of propagation related to motor impairment and medication response. These new insights into basal ganglia function could help improve future neuromodulation strategies.

**See Hart (https://doi.org/10.1093/brain/awag133) for a scientific commentary on this article.**

## Introduction

The cortico-basal ganglia network plays a central role in orchestrating the selection, scaling and adjustment of behavioural output, with the subthalamic nucleus (STN) serving as a key integrative hub for fine-tuning motor function.^1^ Disrupted STN dynamics impair motor control and underlie the pathophysiology of movement disorders.^2-4^ In Parkinson’s disease (PD), motor deficits are associated with exaggerated STN beta activity (13–35 Hz) at rest and impaired beta desynchronization during movement.^5-9^ In contrast, high-gamma activity (HG, 60–90 Hz) increases during movement, which optimizes movement parametrization and is considered pro-kinetic in PD.^10-16^ Movement-related power modulations can expand into higher frequency ranges above HG, yet their functional significance remains poorly understood.^17-22^ This spectral range encompasses high-frequency oscillations (HFOs), subdivided into slow (∼200–300 Hz, SHFO) and fast (∼300–400 Hz, FHFO) components,^23-26^ which exhibit opposite modulation depending upon the dopaminergic state. SHFOs are more prominent in the OFF medication state, whereas FHFOs are elevated in the ON medication state.^23,25^ Moreover, HFOs show temporal coupling with beta activity through phase-amplitude coupling (PAC), which is associated with impaired motor control in PD and modulated by medication and deep brain stimulation (DBS).^24,27,28^ Traditionally, most studies to date have treated movement-related STN oscillations as local, spatially agnostic power modulations over time.^29-31^ Such approaches likely overlook key aspects of the operational dynamics of the basal ganglia. Indeed, at the cortical level, movement encoding in time also unfolds across spatially distributed networks, providing further advantages for fine-tuning behaviour.^32-34^ Whether similar spatial dynamics contribute to movement-related encoding within the basal ganglia remains an open question. Recent advances using directional multi-contact DBS leads that enable more precise anatomical mapping of STN oscillations, revealed the potential importance of the spatial domain.^35,36^ In fact, oscillations from 1 up to 400 Hz exhibit a heterogeneous spectral architecture in PD patients at rest which appears to be linked to clinical-functional areas within the STN.^37^ The most pronounced spectral gradient occurs along the inferior-superior axis, with the beta activity hotspot localized up to 2 mm more superior compared with higher-frequency activity hotspots, such as fast-gamma (FG) or SHFO. Importantly, this spatial organization changes with the degree of consciousness, suggesting a state-dependent functional architecture.^38^

We hypothesize that movement-related synchronization in the human STN has richer facets than currently described. First, synchronization spans a wide range of the high-frequency domain, yet does so through distinct and non-uniform oscillatory dynamics. Second, we anticipate that synchronization is not limited to local power modulations over time but may also evolve spatially and be subject to spatial regulation within the STN. This perspective suggests a previously unrecognized mode of encoding in the basal ganglia, which may link specific spatiotemporal synchronization patterns to altered motor states in PD. To test this hypothesis, we analysed the spatiotemporal properties of movement-related synchronization in the STN using multi-contact DBS leads, which provide high anatomical resolution, in a large cohort of individuals with PD. We then examined how these spatiotemporal dynamics relate to motor impairment and clinical responsiveness to dopaminergic medication. Uncovering spatially organized features of high-frequency activity in the STN, would advance our understanding of subcortical mechanisms underlying behaviour and might inform the development of spatially adaptive neuromodulation strategies.

## Materials and methods

### Clinical characteristics of patients and DBS surgery

**Figure 1:**
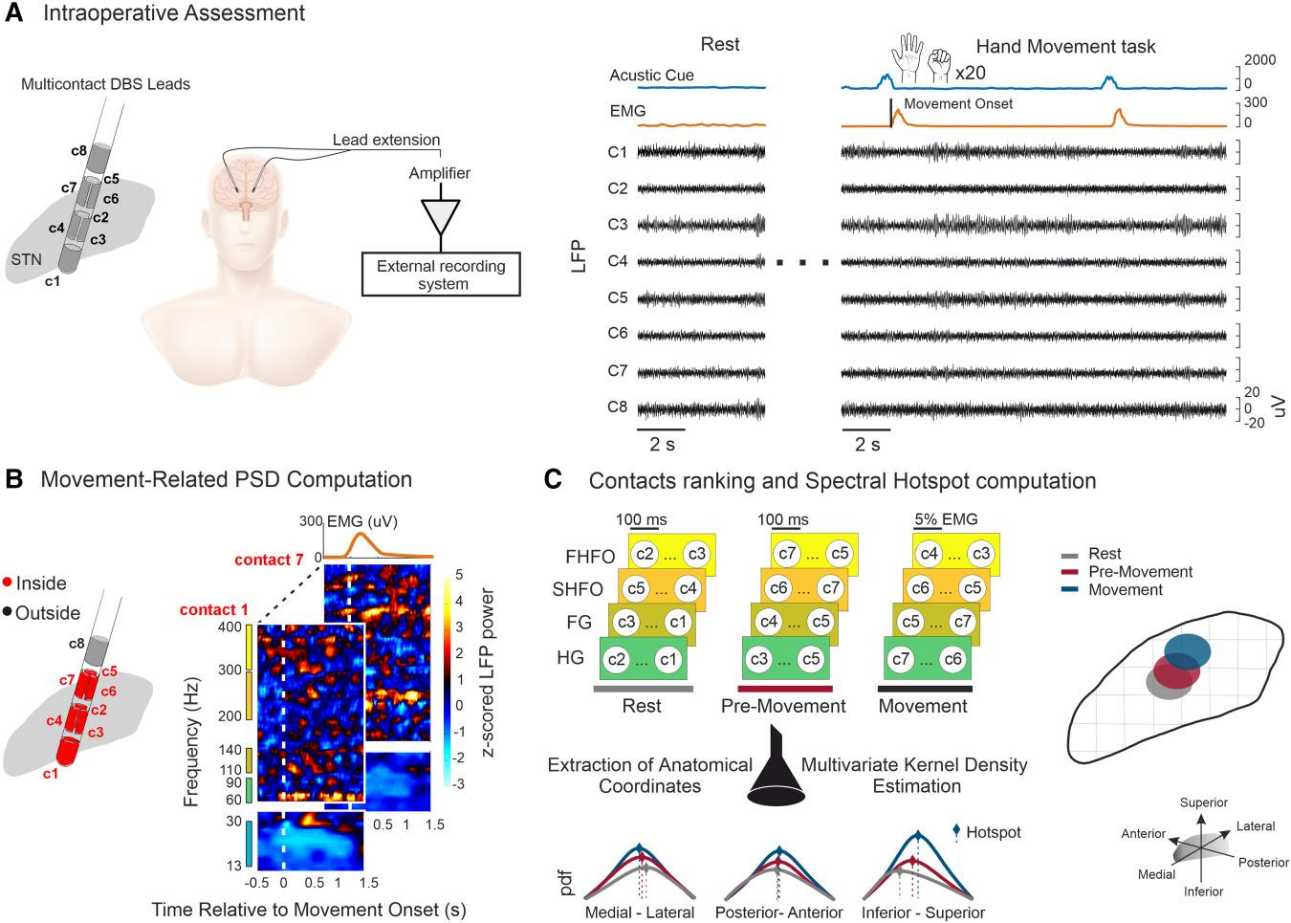
**Overview of data collection and analyses.** (**A**) *Left*: Schematic representation of multi-contact DBS lead implanted in the STN and connected to the intraoperative recording system. *Right*: LFP traces from all eight contacts were recorded in parallel with surface EMG electrodes placed over the forearm flexors. Signals were first recorded at rest (∼1 min) followed by a block of repeated acoustically cued upper limb movements, consisting of hand closure and opening. Each trial was divided in a pre-movement and movement part, with an intertrial interval of 8 s. (**B**) Only contacts localized inside the anatomical STN (coloured in red) were considered for the analyses. Spectrogram illustrates example of normalized movement-related spectral amplitude changes (event-related synchronization, ERS) in high-frequency bands up to 400 Hz extracted from contacts inside the STN of a representative patient. (**C**) *Left*: Contacts were ranked based on their normalized spectral amplitude in each frequency band across rest, pre-movement and movement states, and their corresponding anatomical coordinates [*x*, *y*, *z* in MNI space] were extracted. Spatial probability density distributions of each sub-band were computed across the three anatomical axes during movement (0 to 100%) using a weighted kernel density estimator. The maximum value (dashed lines) corresponds to the centre of the spectral hotspot in space. *Right*: Projections of hotspot probability distributions onto the anatomical space of the STN across motor states for an exemplary frequency band. DBS = deep brain stimulation; ERS = event-related synchronization; FG = fast-gamma; FHFO = fast high-frequency oscillations; HG = high-gamma; LFP = local field potential; MNI = Montreal Neurological Institute; pdf = probability density function; SHFO = slow high-frequency oscillations; STN = subthalamic nuclei.

We screened 46 patients with PD who underwent bilateral STN-DBS surgery at the University Hospital in Bern including a hand motion task in parallel to the intraoperative neurophysiological assessment. Owing to unilateral recordings following intraoperative time constraints, this assessment was available in 77 of 92 hemispheres. From those we further selected hemispheres with at least two stimulation contacts localized inside the normalized STN, resulting in a total of 40 patients and 63 hemispheres which we included for further analyses (Supplementary Table 1). Part of this cohort was previously reported.^36^  ^,37,39-41^ All subjects provided their general consent for biomedical research (local ethics protocol 2017-00551). The cohort comprised 24 male and 16 female patients, with an average age at the time of surgery of 61.1 ± 9.2 years, and an average disease duration of 9.4 ± 4.4 years. Their mean Movement Disorders Society Unified Parkinson’s Disease Rating Scale (MDS-UPDRS) Part III score during the preoperative levodopa challenge was 39.4 ± 11.4 [mean ± standard deviation (SD)] in the OFF state and 14.6 ± 7.6 in the ON state. Based on the predominant clinical phenotype, the cohort consisted of 32 akinetic-rigid (51 hemispheres), two mixed-type (three hemispheres) and six tremor-dominant patients (nine hemispheres). Their average levodopa equivalent daily dose was 1084.2 ± 563.4 mg. Note, the OFF state was assessed more than 48 h after dopamine agonist withdrawal and more than 8 h after levodopa withdrawal. For the ON state, patients received a suprathreshold dose of levodopa, or about 1.5 times their usual morning levodopa equivalent dose. DBS surgery was performed OFF medication. All patients were implanted with the Boston Vercise Cartesia directional leads (Boston Scientific) (Fig. 1A). The DBS target was identified based on the T2-sequence of the preoperative 3 T MRI and preoperative stereotactic CT-scan (Leksell G frame) using Brainlab Elements software (Brainlab AG). Intraoperative targeting was refined through microelectrode recordings and selective test stimulation.

### Intraoperative neurophysiological and behavioural assessment

Local field potentials (LFPs) were recorded after placement of the DBS lead in its final position in the STN using the TMSI-Porti amplifier (Twente Medical Systems International) from all eight contacts simultaneously with a common average reference, the ground electrode placed at the abdomen and a sampling rate of 2048 Hz. First, LFPs were recorded with patients at rest and with eyes open (average duration: 93.24 ± 29.43 s), followed by a hand-motion task with a target goal of 20 contralateral hand closing/opening movements (Fig. 1A). For this, surface EMG electrodes were positioned on the forearm flexor muscles in a belly-tendon montage, with accelerometers on the dorsum of the hand for improved movement detection. Note, each single movement was cued by an auditory signal (verbal ‘go’ command) captured by a microphone synchronized with the recording system. In addition, blocks of ipsilateral movements could have been performed in 11 hemispheres. The average time delay between the auditory cue onset and movement onset was 0.43 ± 0.18 s and the intertrial interval was 9.0 ± 2.67 s.

### Biosignal processing

Spike2 software from CED (Cambridge, UK) was used to manually select the onset of the audio cue and to determine the start and end of movements based on the EMG signal. MATLAB (MathWorks, Natick, MA, USA) custom-made scripts were the used for trial segmentation, artefact cleaning, preprocessing and subsequent analyses. Trials were aligned to the EMG onset, defined as the rising phase of the rectified EMG signal, and extended from 3 s before EMG onset to 2 s after EMG offset, identified by the end of the descending phase. EMG signals were downsampled to 800 Hz, the direct current component was removed, high-pass filtered at 10 Hz and rectified. LFP signals were also downsampled to 800 Hz, high-pass filtered at 0.5 Hz, notch-filtered at 50 Hz and its harmonics (up to 400 Hz). Each individual trial underwent visual inspection, and trials with artefacts were excluded, resulting in an average of 17.93 ± 4.7 trials per motion block.

### Biosignal analyses

The power spectrum was estimated using the multitaper method^42^ with parameters optimized for the frequency range of interest. The spectrum was computed in two steps: for the low-frequency range (10–50 Hz) we used overlapping windows of 400 ms (shifted by 10 ms) with spectral smoothing of ±2 Hz resulting in a frequency resolution of 2.5 Hz. For higher frequencies (50–395 Hz), we used windows of 200 ms (shifted by 10 ms) and spectral smoothing of ±10 Hz, resulting in a frequency resolution of 5 Hz. Power changes in both the pre-movement and movement states were estimated for the following five frequency sub-bands: beta- (13–30 Hz), HG(60–90 Hz), FG(110–140 Hz), SHFO (202–298 Hz) and FHFO (302–390 Hz) (Fig. 1B), calculated by *Z*-scoring relative to a baseline period (−3 to −2 s before movement onset). LFPs were analysed across three behavioural states: rest, pre-movement and movement. The resting state was defined as the patient being relaxed with eyes open without any motor engagement. The pre-movement and movement states were extracted from the single movement trials. During the pre-movement state the patient was already in a readiness state for the upcoming motion and was defined as a 1-s window from −1.5 to −0.2 s before the auditory cue, not overlapping with the baseline period. The movement state corresponds to the execution of the movement following the acoustic cue. Given the intra-individual variability in movement duration (1.54 ± 0.59 s), we performed a percentage normalization of the movement duration (0% onset, 100% end of movement) and further segmented this into 5% bins to align trials for statistical analysis. Cumulative event-related power changes during the movement phase were computed as the area under the curve (AUC) by summing the event-related synchronization (ERS) values over the movement window.

### Localization of DBS contacts and spectral hotspot localization

DBS contact localization was performed postoperatively using the Lead-DBS toolbox in MATLAB.^43^ Preoperative MRI and postoperative CT scans were co-registered and normalized to the Montreal Neurological Institute (MNI) space (MNI152 NLIN 2009b).^44^ Contacts from both hemispheres were projected onto the right STN (DISTAL atlas) using a non-linear flip function. The in-built MATLAB function intriangulation was used to detect contacts inside the STN and their relationship to motor, associative and limbic subregions. The movement-related spectral topography of the STN was determined following a methodology described in a previous publication.^37^ For each DBS lead, the contact inside the STN with the highest event-related power per sub-band was identified (Fig. 1C). Using the MNI coordinates and power values of these contacts, a multivariate kernel density estimation was computed to determine the position of maximum power for each sub-band. Hotspot positions were defined as the peak of the probability density function per axis (Fig. 1C). Note, owing to the small and unbalanced sample sizes in the tremor-dominant and mixed subgroups, hotspot computation included all hemispheres irrespective of the clinical phenotype. To assess the propagation of hotspots, we first quantified the change in their *x*, *y* and *z* coordinates during 100 ms epochs within the resting state and pre-movement state, as well as during 5% epochs during the movement state (Fig. 1C). The positional change between the three states was defined as the average difference in coordinate values of the group-level hotspot and termed spatial propagation. The hotspot volatility was calculated at the individual level by measuring the percentage of time bins in which the contact with the highest ERS activity in each sub-band changed.

### Ranking of temporal and spatial LFP features

To determine the predictive importance of LFP temporal and spatial features for clinical measures (OFF MDS-UPDRS III and % improvement in motor scores), we implemented a least squares boosting (LSBoost) model using MATLAB’s *fitrensemble*. This method is suited to model complex, non-linear relationships while minimizing overfitting.^45^ Hyperparameters (*LearnRate* and *NumLearningCycles*) were optimized within the ranges (0.3, 0.6) and (30, 200), respectively, using the *OptimizeHyperparameters* option. Feature importance was assessed with the *predictorImportance* function, ranking features based on their contribution to reducing prediction error across boosting iterations. Model performance was evaluated using leave-one-out cross-validation (LOOCV), where the model was trained on all but one sample and tested on the remaining sample, iterating this process across all data-points. Hyperparameters were optimized separately on each LOOCV fold to avoid data leakage. Prediction accuracy was quantified by computing the Pearson’s correlation between predicted and actual outcomes, along with the root mean squared error (RMSE).

### Statistical analyses

Statistical analyses were performed using MATLAB (2022b; Mathworks, Natick, MA, USA). The event-related power changes were analysed using a one-way repeated measure ANOVA with frequency bands being the within-subject factor followed by a Tukey’s *post hoc* test. To compare the contralateral versus ipsilateral ERS we used a two-way repeated measures ANOVA with frequency bands as within-subject factor and recording side (contralateral/ipsilateral) as a between-subjects factor. The paired Wilcoxon signed-rank test was used to determine the differences between time to maximum ERS across the different frequency bands and between the frequency bands and EMG. To assess whether the spectral hotspot locations differed from chance, we compared them to the 5% critical values (two-tailed) of random surrogate distribution hotspot coordinates. To assess whether the propagation of hotspots differed from chance, we compared the observed propagation to the 5% critical values (two-tailed) of surrogate propagation values (*n* = 1000). These surrogate distributions were generated in two steps: first, we computed 1000 random hotspots, by randomly selecting contacts within each hemisphere during the movement state. Secondly, we computed the spatial difference between the actual pre-movement hotspot and the random hotspot distribution. Spearman’s correlation coefficient was used for all the correlation analysis. Confidence intervals (95%) were estimated using a bootstrap method with 5000 resamples. To evaluate the statistical reliability of a feature’s importance, we tested it against a null distribution of importance scores (*n* = 1000), calculated by randomly shuffling the clinical measure values (OFF MDS-UPDRS III and % improvement in motor scores). All significant *P*-values were corrected for multiple comparisons using the false discovery rate (FDR) method.

## Results

### Temporal dynamics of movement-related oscillations

**Figure 2:**
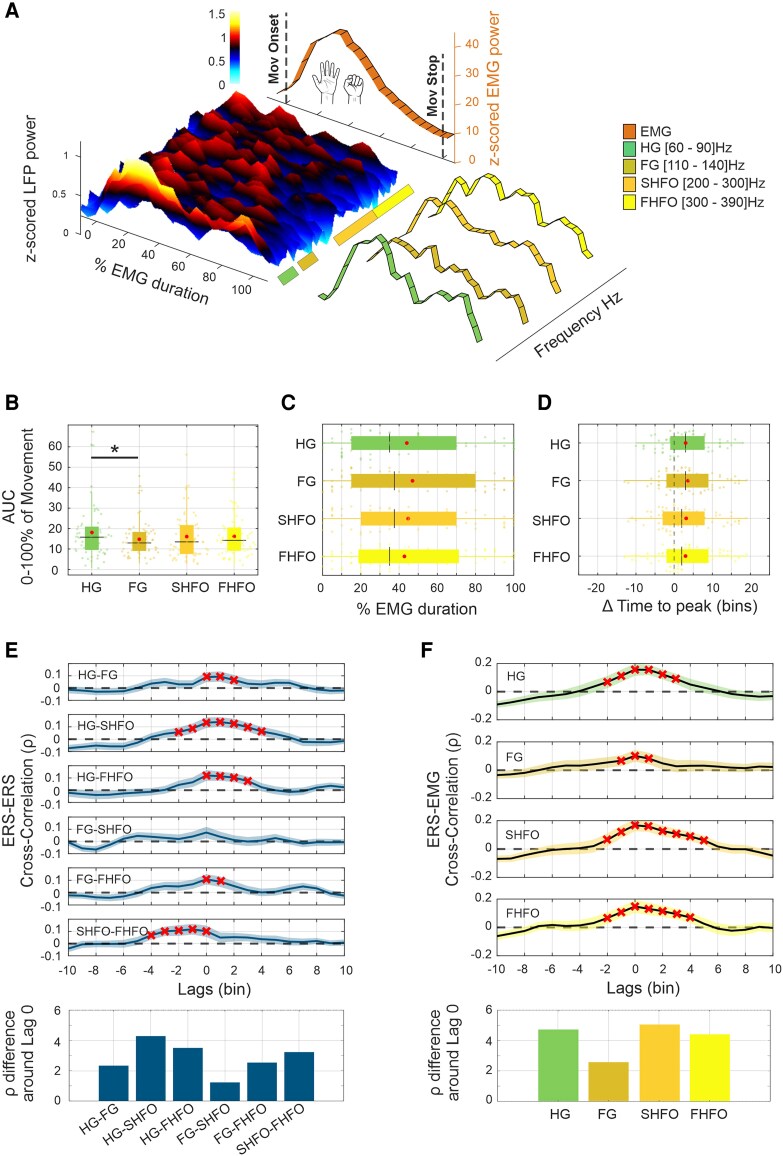
**Magnitude and temporal dynamics of movement-related synchronization.** (**A**) Oscillatory spectral power changes occur across all high frequencies during voluntary contralateral hand movements. Mean power changes, aligned to movement onset (0% of the movement phase) were grouped by bands: HG, FG, SHFO and FHFO. (**B**) Total ERS, measured as area under the curve (AUC) (0 to 100% of movement), for each sub-band and across hemispheres. HG showed significantly greater synchronization compared with FG. (**C**) Average timing of peak ERS per hemisphere relative to the 0% to 100% of movement with no significant differences across frequency bands. (**D**) Time lag between the peak of EMG activity and the peak of ERS across hemispheres. All frequency bands reached their ERS peaks with a significant delay after the EMG peak: HG (Δ_ERS-EMG_ = 15.01 ± 33% mean ± SD, *P*_FDR_ < 0.01), FG (Δ_ERS-EMG_ = 19 ± 35.9%, *P*_FDR_ <0.01), SHFO (Δ_ERS-EMG_ = 15.3 ± 39%, *P*_FDR_ =0.01) and FHFO (Δ_ERS-EMG_ = 14.9 ± 37.5% *P*_FDR_ <0.01). Red dots and black lines represent the mean and median of distributions, respectively. (**E**) Cross-correlation of sub-band time courses (*top*) and mean standardized difference ±3 bins around lag 0 (*bottom*). Distinct dynamics were observed across sub-bands. Shaded areas represent ± standard error of the mean (SEM). (**F**) Cross-correlation between band-specific ERS and EMG activity (*top*) and mean standardized difference ± time bins around lag 0 (*bottom*). Significant coupling, yet with a different temporal extent, was found for HG, FG, SHFO and FHFO, with peak correlations centred around 0 lag. Shaded areas indicate ± SEM. ERS = event-related synchronization; FDR = false discovery rate; FG = fast-gamma; FHFO = fast high-frequency oscillations; HG = high-gamma; SHFO = slow high-frequency oscillations.

Intraoperative LFPs from STN were analysed from 63 hemispheres of 40 patients with PD using high-resolution multi-contact DBS leads (Fig. 1A and Supplementary Table 1). Recordings were obtained with the patient awake, OFF dopaminergic medication and both at rest and during blocks of multiple and distinctly cued movements of the contralateral hand (opening/closing) separated into a pre-movement and movement period (Fig. 1A). Here, we first report and contrast the movement-related spectral power modulation for each sub-band (HG, FG, SHFO, FHFO; in totalspanning from 60 to 400 Hz) and corresponding DBS lead contact evidencing the strongest synchronization (Fig. 1B). During voluntary movement, the spectral analysis showed a power increase that extends upon the entire frequency span (Fig. 2A). The cumulative ERS power across frequency bands, computed as AUC, was statistically stronger for HG compared with FG (*P*_Tukey’s_ = 0.01), but without differences between the other sub-bands (Fig. 2B) or across clinical phenotypes (Supplementary Fig. 1). The muscle contraction peaked on average at approximately 29% of the movement (∼0.43 s after movement onset) while the synchronization maxima were reached later at 44% of the movement evolution (∼0.67 s after movement onset) for HG, 48% (∼0.73 s) for FG, 44% (∼0.68 s) for SHFO and at 43% (∼0.64 s) for FHFO (Fig. 2C), with significant differences for all sub-bands in the EMG-ERS peak intervals (Fig. 2D). When contrasting the temporal evolution of sub-band-specific ERSs against each other, differences in timing and correlative strength were evident during the movement. Specifically, FG shared less common temporal properties compared with the other bands and SHFO preceded FHFO, with peak correlation at lag −1 (∼5% of movement duration) (Fig. 2E). When comparing the temporal dynamics of movement-related synchronization to forearm EMG activity, we found a significant cross-correlation predominantly centred at lag 0, with the strongest and most sustained coupling observed for HG, SHFO and FHFO (Fig. 2F). A subgroup of patients also performed a movement task using the hand ipsilateral to the recorded STN (Supplementary Fig. 2). The ERS magnitude did not significantly differ between ipsilateral and contralateral hemispheres and the timing of peak synchronization was comparable. Within the ipsilateral group, no significant temporal correlations were found between spectral sub-bands. Moreover, the ipsilateral ERS showed a weaker and different cross-correlation to EMG activity, with HG showing an inverse correlation preceding muscle activity, while FG followed EMG activation. No significant correlations were found for SHFO and FHFO.

**Figure 3:**
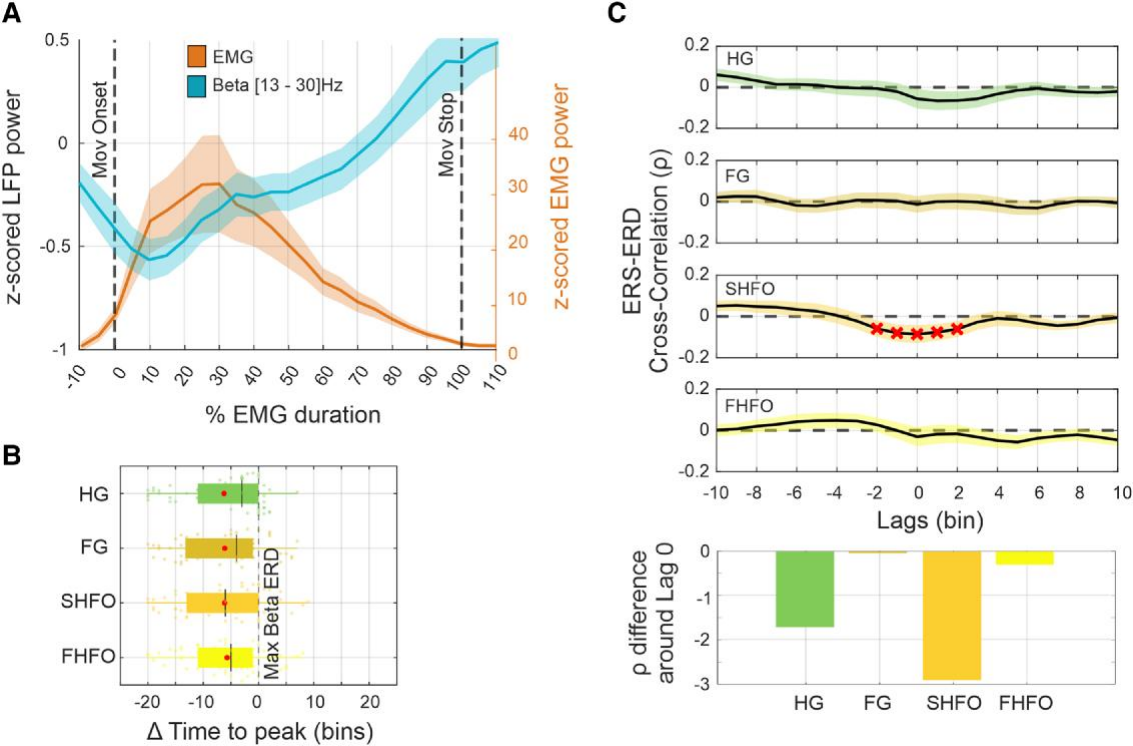
**Temporal dynamics of movement-related beta desynchronization and high-frequency synchronization.** (**A**) Oscillatory spectral power changes in the beta band (13–30 Hz) during voluntary contralateral hand movements, aligned to the normalized movement onset (0% of the movement phase). (**B**) Comparison of timing between peak in beta ERD and peak ERS in high-frequency bands across hemispheres. Beta ERD peaks significantly earlier than ERS evident for all sub-bands. HG (Δ_ERS-ERD_ = −30.9 ± 37.1% mean ± SD, *P*_FDR_ < 0.001), FG (Δ_ERS-ERD_ = −31.05 ± 37.7%, *P*_FDR_ <0.001), SHFO (Δ_ERS-ERD_ = −29.82 ± 39.2%, *P*_FDR_ <0.001) and FHFO (Δ_ERS-ERD_ = −28.04 ± 34.81%, *P*_FDR_ < 0.001). (**C**) Cross-correlation between beta ERD and high-frequency ERS dynamics (*top*) and mean standardized difference ±3 bins around lag 0 (*bottom*). A specific and significant inverse relationship was observed between beta and SHFO, while no coupling was found for HG, FG and FHFO. Shaded areas represent ± standard error of the mean. ERS = event-related synchronization; FDR = false discovery rate; FG = fast-gamma; FHFO = fast high-frequency oscillations; HG = high-gamma; SHFO = slow high-frequency oscillations.

Consistent with results previously reported from non-invasive and invasive human electrophysiology studies,^46-49^ we observed maximum event-related desynchronization (ERD) in the beta band (13–30 Hz) preceding the EMG peak and followed by a rebound synchronization after the end of the movement (Fig. 3A and Supplementary Fig. 3). Contrasting the ERD- to the ERS-dynamics we observed that the maximum beta-ERD (at around 14%, ∼0.22 s after movement onset) consistently preceded the ERS maxima to a similar extent for all sub-bands (Fig. 3B). Interestingly, differences were found when assessing the time-magnitude relationship throughout the entire motion, as the beta ERD shows an exclusive and inverse correlation with the SHFO-ERS for the contralateral STN (Fig. 3C), while a different band-specific ERS-ERD relationship was evident for the ipsilateral STN (Supplementary Fig. 2D).

### Spatial dynamic of movement-related oscillations

**Figure 4:**
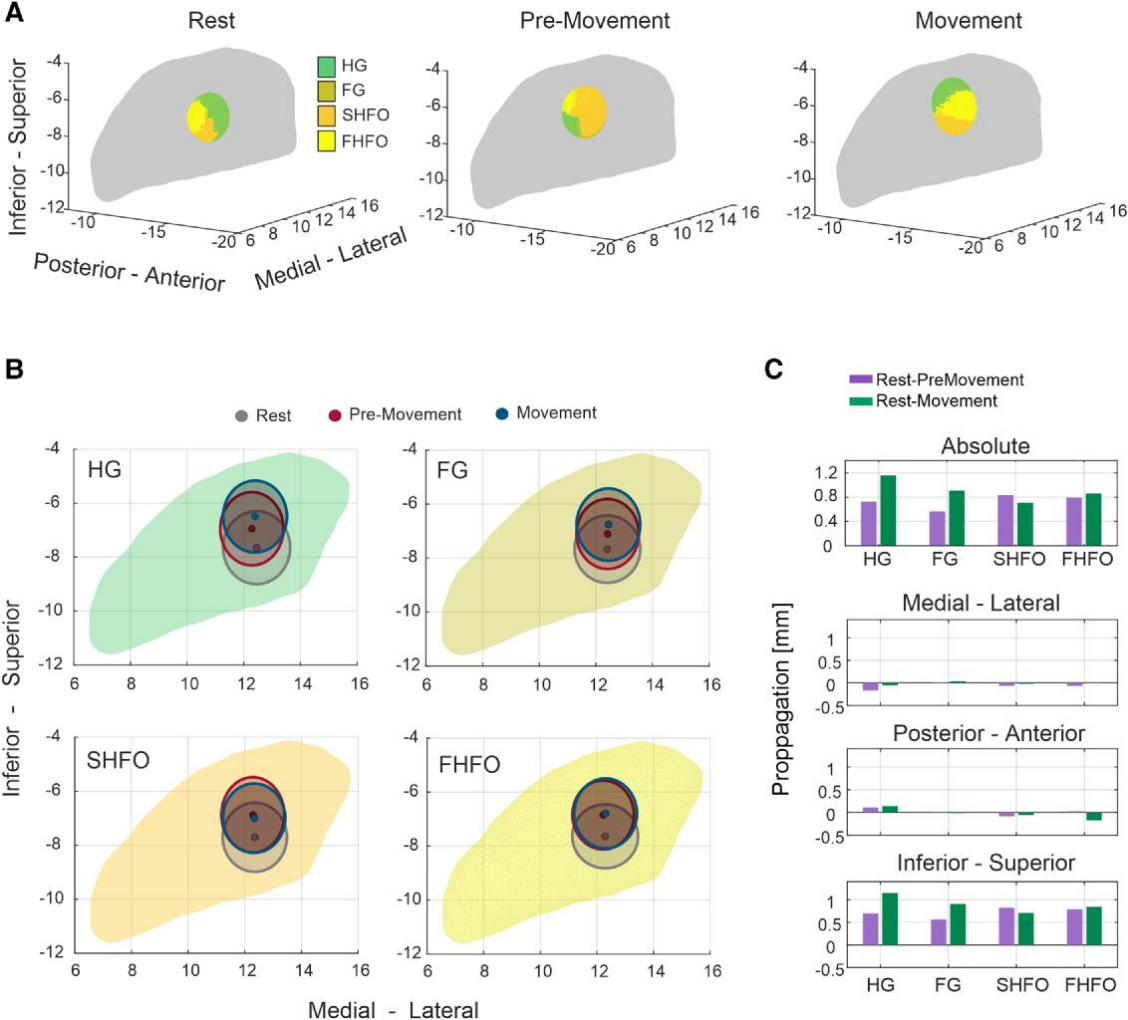
**Spatial hotspot dynamics of high-frequency activity from resting to movement state.** (**A**) 3D probabilistic representation of spectral activity (HG, FG, SHFO and FHFO) within the anatomical boundaries of the STN during rest, pre-movement and active movement phases. Each 3D ellipsoid represents the spatial distribution of a band-specific hotspot, with the centre corresponding to the peak of distributions and the ellipsoid borders reflecting the mean absolute distance between the hotspot centre and all coordinates generated by the kernel density estimation. (**B**) 2D projections of hotspot probability distributions onto the inferior-superior and medial-lateral axes across motor states, grouped by frequency band (HG, FG, SHFO, FHFO). (**C**) Mean spatial propagation (*x*, *y*, z coordinates) of peak spectral activity across sub-bands during motor state transitions, indicating dynamic reorganization of STN activity, primarily along the superior-inferior axis. FG = fast-gamma; FHFO = fast high-frequency oscillations; HG = high-gamma; SHFO = slow high-frequency oscillations; STN = subthalamic nucleus.

**Figure 5:**
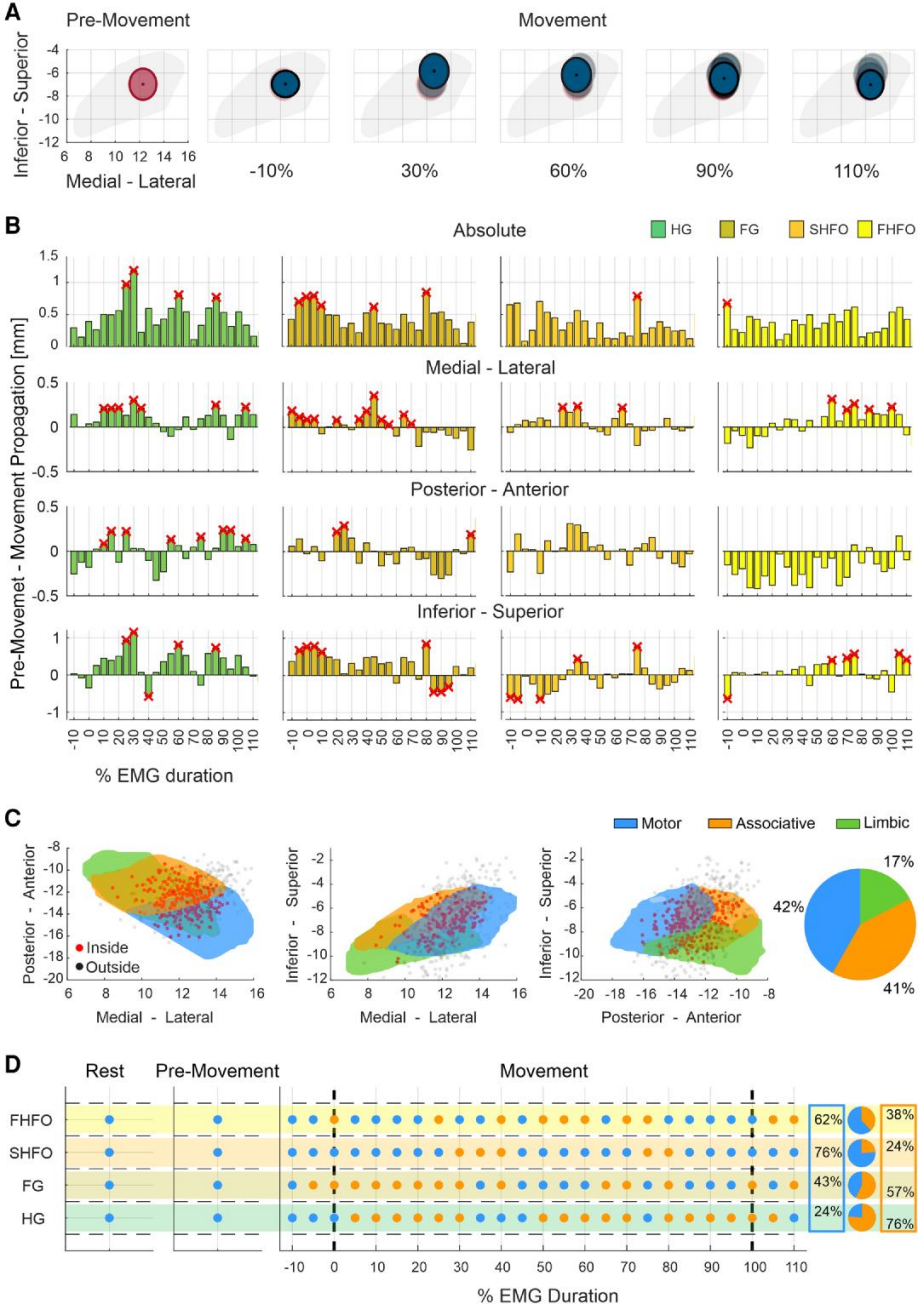
**Spatio-temporal dynamics of motion-related neuronal synchronization.** (**A**) 2D probabilistic representation of a representative spectral hotspot propagation within the STN during pre-movement and at different steps of the movement phase: i.e. −10%, 30%, 60%, 90% and 110%. Each ellipse represents the hotspot probability distribution: the centre indicates the peak of the distribution, and the borders reflect the mean absolute distance between the hotspot centre and all coordinates generated by the kernel density estimation. (**B**) Bar plots showing temporal evolution of hotspot propagation by sub-band across the medial-lateral, anterior-posterior and inferior-superior axes, and the absolute Euclidean distance. HG and FG show significant propagation towards superior STN at multiple time points. In contrast, SHFO shows a tendency towards inferior displacement, while FHFO shows no clear directional preference. Red crosses indicate FDR-corrected significant time points. (**C**) 3D scatter plot of the contacts of the entire cohort inside (red) and outside (black) the STN in MNI space. The STN is shown according to its tripartite subdivision (blue: motor, orange: associative, green: limbic). The pie chart indicates the fraction of contacts in each subregion, showing that most contacts are located within motor and associative territories. (**D**) Subregion analysis of spectral hotspots across rest, pre-movement and at different time points during the movement phase for each frequency band. The pie chart shows the fraction of hotspot positions within the three STN subregions during movement. HG and FG hotspots dynamically shift between motor and associative regions during movement, while SHFO and FHFO remain largely confined to the motor STN. FDR = false discovery rate; FG = fast-gamma; FHFO = fast high-frequency oscillations; HG = high-gamma; MNI = Montreal Neurological Institute; SHFO = slow high-frequency oscillations; STN = subthalamic nucleus.

The high-resolution multi-contact DBS leads used in this work allow us to investigate the presence and direction of spatial activity shifts within the STN in the context of motor activation. For this we computed the position (*x*, *y*, *z* coordinates) of the hotspots for the different spectral sub-bands during the three distinct states: resting-, pre-movement- and movement-state which are illustrated in Fig. 4A and reported in Supplementary Table 2. These spatial dynamics were quantified as absolute and axis-specific changes. Already with the transition from the resting state to the pre-movement state we observed an evident spatial propagation of spectral hotspots within the STN most pronounced along the inferior-superior axis (Fig. 4B and C). An even more evident spatial propagation was observed during the transition from the resting to the movement state, again predominantly along the inferior-superior axis (Fig. 4C). We then analysed the evolution of hotspot propagation at different percentage time points around the movement period, with the pre-movement period hotspot location as comparator (Fig. 5A). HG showed a significant propagation at multiple time points along the *x*-axis, *y*-axis and *z*-axis, reaching a maximum propagation of 1.18 mm in the superior direction. Similarly, FG showed multiple significant hotspot propagations along the three axes, with a maximum propagation of 0.84 mm in a superior direction. Both SHFO and FHFO showed weaker and directionally different propagation patterns, with significant propagations along the *x*-axis and *z*-axis, no changes along the *y*-axis and with an overall tendency towards the inferior direction for SHFO (Fig. 5B). Noteworthy, a similar propagation pattern was also observed at the individual level (Supplementary Fig. 4).

Of further interest is how hotspots and their propagation are localized relative to the functional subregions of the STN. The contacts used to identify hotspot positions were spread across motor, associative and limbic territories, with the majority located in the dorsal STN, consistent with the surgical strategy (Fig. 5C). During the rest and pre-movement state, hotspots of all sub-bands were located within the motor region. Interestingly, during movement HG and FG hotspots dynamically shifted between motor and associative regions, while both SHFO and FHFO remained largely confined to the motor part (Fig. 5D).

**Figure 6:**
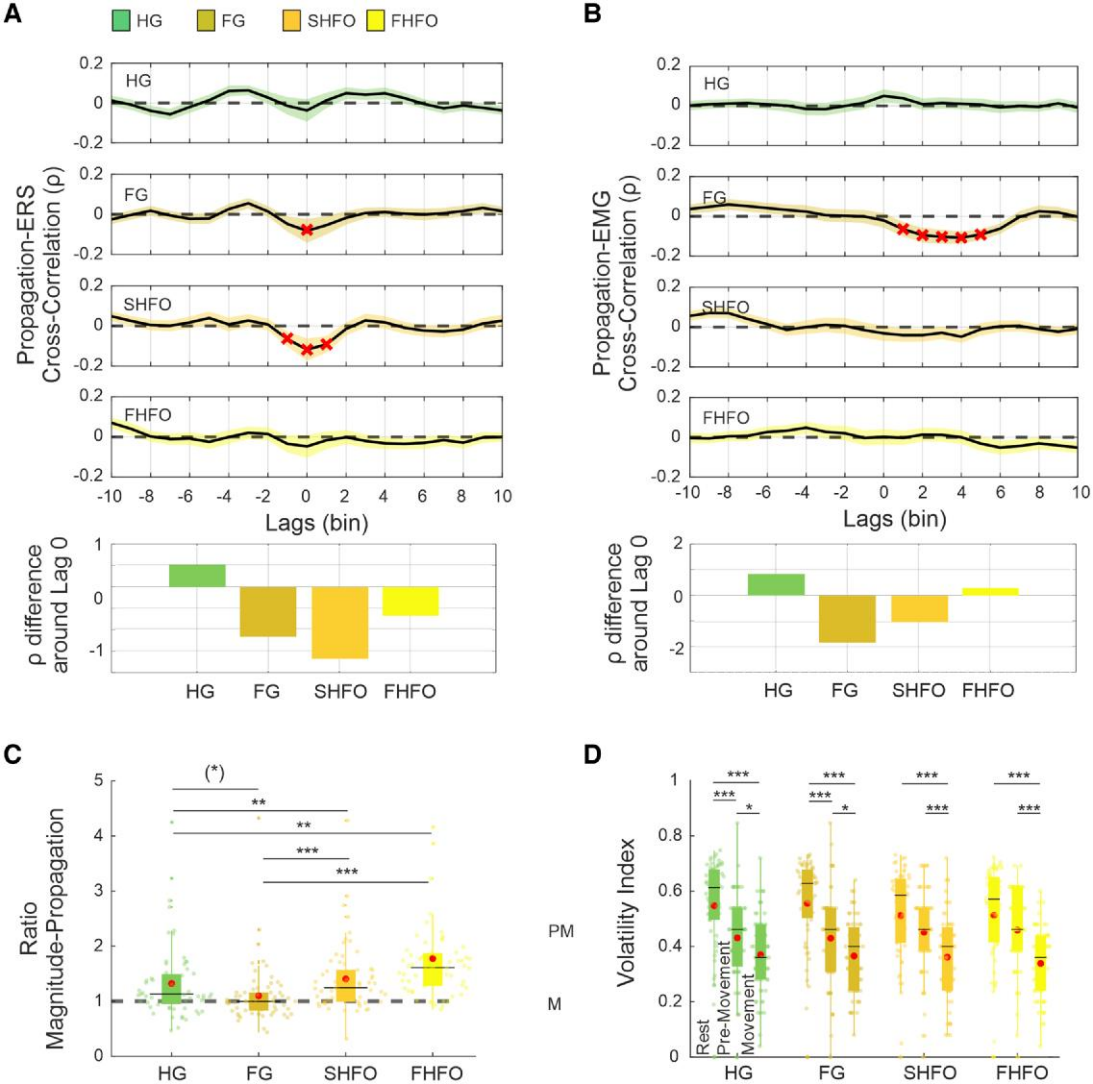
**Characteristics of spatiotemporal dynamics.** (**A**) Cross-correlation between sub-band specific ERS amplitude and hotspot propagation. *Top*: Cross-correlation traces; *bottom*: mean standardized differences (±3 time bins) around lag 0. FG and SHFO exhibit significant inverse correlations, suggesting decoupling between spatial shifts and spectral magnitude, while HG and FHFO show no temporal coupling. Shaded areas represent ± standard error of the mean. (**B**) Cross-correlation between sub-band-specific hotspot propagation and EMG activity. *Top*: Cross-correlation traces; *bottom*: standardized difference bars (±3 time bins) around lag 0. FG shows a significant inverse relationship, with propagation preceding muscle activation. (**C**) Magnitude-propagation ratio across pre-movement and movement states. Each dot represents one hemisphere. FG exhibits a relatively lower power-propagation ratio compared with other sub-bands, indicating proportionally greater spatial displacement. HG = 1.3 ± 0.59 mean ± SD, *P*_FDR_ < 0.001, FG = 1.11 ± 0.61, *P*_FDR_ = 0.989, SHFO = 1.63 ± 0.62, *P*_FDR_ < 0.001 and FHFO = 1.73 ± 0.85, *P*_FDR_ < 0.001. (**D**) Analysis of hotspot location volatility during rest, pre-movement and movement states. Each dot indicates the frequency of positional shifts per hemisphere. All sub-bands showed reduced volatility during movement states compared with rest at both the individual and group level. *P < 0.05; **P < 0.01; ***P < 0.001. ERS = event-related synchronization; FDR = false discovery rate; FG = fast-gamma; FHFO = fast high-frequency oscillations; HG = high-gamma; SHFO = slow high-frequency oscillations.

Next, we investigated whether the spatial propagation is associated with the magnitude of neural synchronization. We found that this is not necessarily the case. Although HG exhibited both the largest average propagation and the greatest ERS magnitude, the cross correlation did not reveal any temporal relationship between the two metrics. However, a significant negative correlation was found for SHFO and FG (Fig. 6A). The latter also showed a significant inverse relationship to the EMG, with the propagation of FG even preceding muscular (Fig. 6B).

We then assessed whether the investigated sub-bands differed in their proportion of synchronization manifesting as power increase or as spatial propagation by comparing the ratio between these synchronization metrics. First, all sub-bands except FG showed a significantly positive ratio, meaning that the magnitude of synchronization was greater than the propagation in space. Across sub-bands, this ratio was significantly higher for SHFO (*P*_Tukey’s_ < 0.01) and FHFO (*P*_Tukey’s_ < 0.01) compared with HG and FG (Fig. 6C).

Finally, we assessed the hotspot volatility at the individual level during the resting, pre-movement and movement states by calculating the percentage of time spent switching between contacts showing the highest ERS activity. Volatility was highest at rest and significantly decreased towards the movement state across all hemispheres, with no significant differences between sub-bands (Fig. 6D).

### Temporal-spatial dynamics and parkinsonian motor states

**Figure 7:**
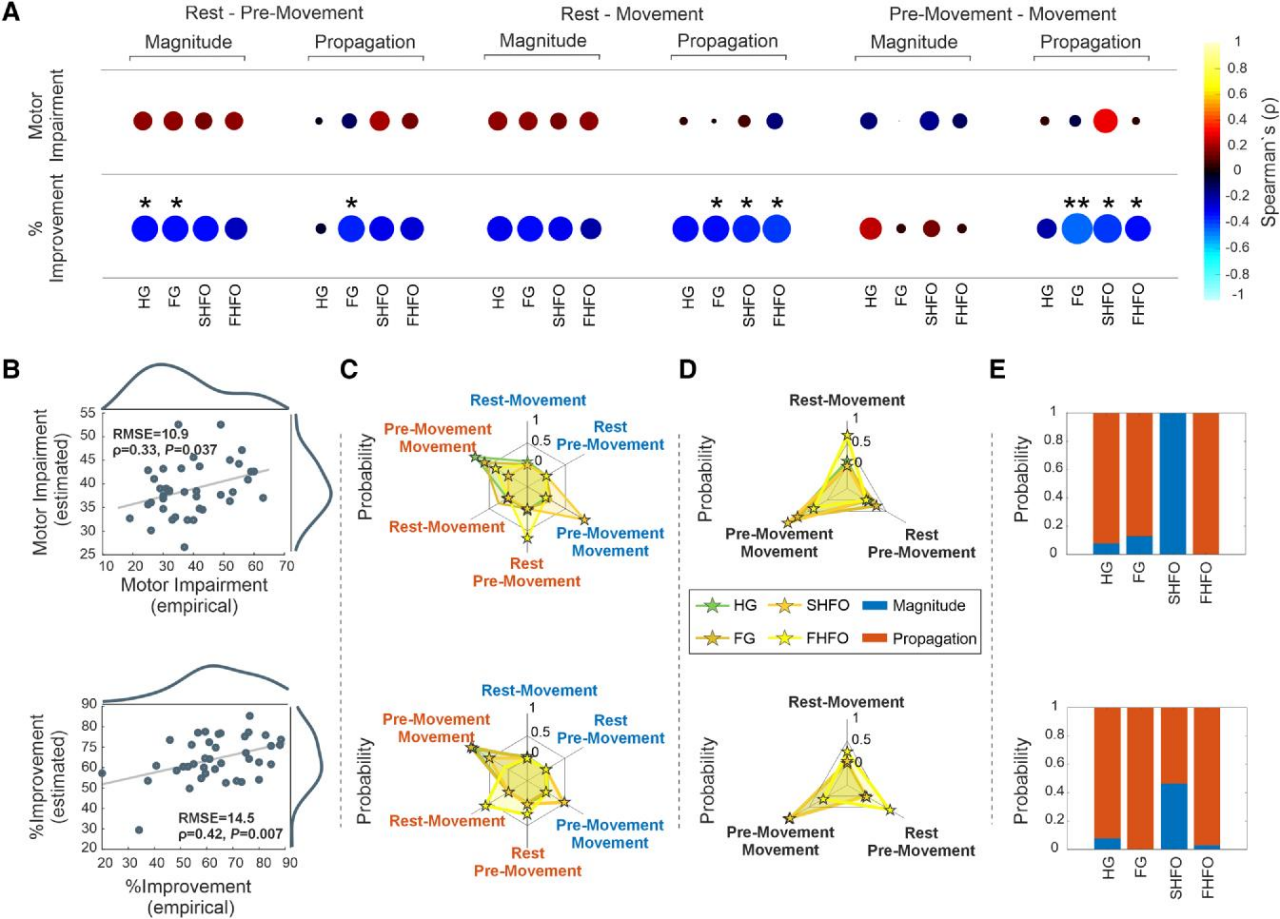
**Relationship between clinical motor state with spatial and magnitude synchronization metrics.** (**A**) Spearman’s correlation analysis between ERS features—magnitude and spatial propagation—and clinical motor outcomes across three motor state transitions: rest-to-pre-movement, rest-to-movement and pre-movement-to-movement. The *top row* shows correlations with motor impairment in the OFF medication state, while the *bottom row* shows correlations with percentage motor improvement following dopaminergic medication. Bubble size and colour represent Spearman’s ρ values; significant correlations are indicated by asterisks. Both magnitude and spatial ERS features showed no significant correlation with motor impairment in the OFF medication state. Greater spatial propagation in FG [ρ = −0.339, *P* = 0.032, 95% CI (−0.62, −0.02)], SHFO [Spearman’s ρ = −0.364, *P* = 0.021, 95% CI (−0.61, −0.07)] and FHFO [ρ = −0.386, *P* = 0.014, 95% CI (−0.61, −0.92)] during rest-to-movement, in FG during rest-to-pre-movement [ρ = −0.347, *P* = 0.028, 95% CI (−0.61, −0.05)], and in FG [ρ = −0.461, *P* = 0.003, 95% CI (−0.70, −0.15)], SHFO [ρ = −0.396, *P* = 0.011, 95% CI (−0.63, −0.10)] and FHFO [ρ = −0.318, *P* = 0.046, 95% CI (−0.57, −0.01)] during pre-movement-to-movement were significantly correlated with reduced motor improvement. Additionally, ERS power in HG [ρ = −0.334, *P* = 0.038, 95% CI (−0.60, −0.01)] and FG [ρ = −0.333, *P* = 0.038, 95% CI (−0.61, −0.005)] during the rest-to-pre-movement phase were significantly negatively correlated with motor improvement. (**B**) LSBoost regression models (leave-one-out cross-validation) accurately predicted both motor impairment (*top*) and % improvement (*bottom*), with significant correlations between actual and predicted values. (**C**) Spider plot showing the ranking of the predictor variable importance (0%–100%). *Top*: Probability that sub-band features (ERS power or propagation) within (rest-pre-movement, rest-movement, pre-movement-movement) emerged as the top-ranked predictor of motor impairment. *Bottom*: Same computation for the prediction of % improvement. Spatial propagation of HG and FG and ERS power of SHFO during the pre-movement-to-movement state were the most predictive features for both motor impairment and improvement, while FHFO propagation was the most predictive during rest-to-movement (impairment) and rest-to-pre-movement (improvement). (**D**) *Top*: Spider plot showing probability that sub-band features within (rest-pre-movement, rest-movement, pre-movement-movement) emerged as the top-ranked predictor of motor impairment. *Bottom*: Same computation for the prediction of percentage improvement. The pre-movement-to-movement state best predicted motor impairment and improvement in HG, FG and SHFO, while FHFO showed the strongest predictions during rest-to-movement and rest-to-pre-movement transitions. (**E**) Bar plot showing probability that sub-band features (ERS power or propagation) emerged as the top-ranked predictor of motor impairment. Motor impairment predictions were most strongly linked to spatial propagation in HG, FG and FHFO, while ERS power magnitude predicted best in SHFO. For percentage improvement, spatial propagation dominated across all bands. *P < 0.05; **P < 0.01; ***P < 0.001. CI = confidence interval; ERS = event-related synchronization; FG = fast-gamma; FHFO = fast high-frequency oscillations; HG = high-gamma; SHFO = slow high-frequency oscillations. .

Lastly, we investigated whether the magnitude and spatial extent of synchronization during movement are rather physiological phenomena or whether they may also be related to motor pathophysiology in PD. For this we analysed their relationship to the overall motor impairment (MDS-UPDRS-III motor scores OFF levodopa medication) and motor improvement (% change MDS-UPDRS-III motor scores after levodopa intake) with the sub-band-specific magnitude and propagation averaged during the different states (i.e. rest, pre-movement, movement). While the magnitude or spatial ERS features showed a positive correlative trend with motor impairment in the OFF medication state, they did not reach statistical significance (Fig. 7A). A different pattern of relationships emerged when considering the improvement in motor performance following levodopa intake as a clinical metric. In general, we observed that a larger extent of spatial propagation was significantly associated to less motor improvement induced by dopaminergic medications, which was evident in various sub-bands (Fig. 7A). These observations remained significant after bootstrapping for FG during the transition from rest to pre-movement; for FG, SHFO and FHFO from rest-to-movement; and for FG and FHFO from pre-movement to movement. The magnitude of ERS was only indicative in HG and FG between rest and pre-movement states (Fig. 7A).

To support this spectro-clinical link we assessed the predictive value of each sub-band for the patient’s motor state or percentage improvement of motor score. Two separate machine learning models, using an LSBoost algorithm with LOOCV, incorporating all spectral features, were used. Both models demonstrated high predictive power, showing significant correlations (Pearson’s ρ = 0.32, *P* = 0.04 for MDS UPDRS OFF and Pearson’s ρ = 0.42, *P* = 0.008 for % improvement) between actual and predicted outcomes, along with relatively low error (RMSE = 10.9 for MDS UPDRS OFF and RMSE = 14.5 for % improvement) (Fig. 7B). These models were used to rank the ERS-related features based on their predictive weights, reflecting each feature’s contribution to the overall model accuracy, as illustrated in Supplementary Fig. 5.

Finally, we assessed the likelihood of sub-band-specific features to emerge as top-ranked predictors of motor impairment or percentage improvement. For both motor impairment and percentage improvement, the most predictive features were spatial propagation of HG and FG and ERS power of SHFO during the pre-movement to movement state, while FHFO propagation was most predictive during the rest-to-movement phase for impairment and the rest-to-pre-movement phase for improvement (Fig. 7C). Transition-wise, the pre-movement-to-movement phase consistently yielded the best predictions for both motor impairment and percentage improvement in HG, FG and SHFO, while the best predictors in FHFO were during rest-to-movement and rest-to-pre-movement transitions, respectively (Fig. 7D). When comparing feature types, predictions of motor impairment were best associated with spatial propagation in HG, FG and FHFO, whereas ERS power was the top predictor in SHFO. For percentage improvement, spatial propagation again emerged as the dominant feature across all bands (Fig. 7E).

## Discussion

In this work we dissect the spatiotemporal characteristics of movement-related synchronization in the STN, and we characterize how these dynamics evolve over time, propagate in space and relate to PD pathophysiology. To this end, we recorded LFPs from 63 STNs using high-resolution multi-contact DBS leads and investigated the different spectral sub-bands of an extended frequency range up to 400 Hz. We found that synchronization manifests across this broad range of frequencies during active hand movement in both contra- and ipsilateral STN, but that distinct sub-bands exhibit differently weighted magnitudes and temporal profiles. Importantly, movement-related synchronization is not confined to a fixed location within the STN, but is characterized by spectral hotspots that diversely propagate along a common anatomical trajectory and between STN subregions. This propagation is unrelated to the magnitude of the synchronization, suggesting that both magnitude and spatial properties convey complementary facets of motor and associative processing. Notably, exaggerated spatial propagation was found to correlate with reduced improvements in motor symptoms following dopaminergic medication, likely reflecting a compensatory network adaptation. Taken together, our results provide evidence for spatial encoding of movement-related synchronization within the STN and establish a new investigational domain for neural dynamics underlying motor behaviour and pathophysiology of PD (Table 1).

**Table 1:** Qualitative summary table of main findings

	HG	FG	SHFO	FHFO
**Temporal dynamics of movement-related oscillations**				
ERS magnitude	↑↑↑	↑	↑↑	↑↑
ERS–EMG relationship	↑↑↑	↑	↑↑↑	↑↑
ERS–ERD relationship	–	–	↓↓↓	–
**Spatial dynamic of movement-related oscillations**				
Absolute hotspot propagation	↑↑↑	↑↑	↑↑	↑
Dominant direction of hotspot propagation	Superior	Superior	Inferior	–
STN subregions localization rest	Motor	Motor	Motor	Motor
STN subregions localization pre-movement	Motor	Motor	Motor	Motor
STN subregions localization movement	Motor ↔ associative	Motor ↔ associative	Motor	Motor
Propagation–ERS relationship	–	↓	↓↓↓	–
Propagation–EMG relationship	–	↓↓↓	–	–
**Spatial and temporal dynamics and parkinsonian pathophysiology**				
Magnitude–motor impairment relationship	–	–	–	–
Magnitude–% improvement relationship	–	–	–	–
Hotspot propagation–motor impairment relationship	–	–	–	–
Hotspot propagation–% improvement relationship	–	↓↓↓	↓↓↓	↓↓

‘↑’ to ‘↑↑↑’ represent the strength of the observed effect, ranging from weak to strong; ‘↑/↓’ indicate the direction of the effect (increase or decrease, respectively), while ‘–’ denotes no detectable effect; ‘↔’ indicates a bidirectional shift of propagation. Note, hotspot propagation reported in the table refers to transitions from the pre-movement-to-movement phase, unless otherwise specified. ERS = event-related synchronization; ERD = event-related depression; FG = fast-gamma; FHFO = fast high-frequency oscillations; HG = high-gamma; SHFO = slow high-frequency oscillations; STN = subthalamic nucleus.

.

### Movement-related synchronization is broad yet spectrally distinct

The neurophysiology of movement encoding in the high-frequency domain follows a reproducible temporal sequence, wherein movement-related synchronization across all the studied sub-bands consistently follows the peak of muscle contraction and the maximal movement-related beta desynchronization. Within this orchestra of oscillations, synchronization dynamics in HG have been well described to fine-tune kinetics of ongoing movements.^10,11,39^ In the present work, we expand this view and consider frequency dynamics up to 400 Hz. Close examination of ERS revealed that different sub-bands exhibit differently weighted spectral properties, and thus likely hold specific functional roles. Concretely, HG exhibits maximal synchronization magnitudes, specifically in comparison to FG activity. FG is also characterized by a weaker relationship to muscle activity, despite both sub-bands arising from similar neuronal circuits.^50^ This supports that FG likely adds to motion processing in an indirect manner by modulating related cognitive attentional processes, probably by involving an associative functional sub-loop of the STN,^20,51^ which is likely considering its propagation behaviour shown in our results. In contrast, HFO may arise from synchronized local neuronal firing with their frequencies determined by the inter-spike intervals.^17,19^ Functionally, HFO are thought to contribute to motor processing by regulating neuronal activity at high spatial and temporal resolution, but are reportedly altered in PD.^22,23,25^ Indeed, we found that SFHO is the only sub-band exhibiting a temporal relationship with beta-ERD during contralateral movements. Since beta dynamics are also altered in the OFF levodopa state, during which the recordings took place,^8,52,53^ this inverse relationship is probably driven by the neurodegenerative state in PD and echoes findings described for PAC between these bands.^25,27,28,54^

Movement-related synchronization is not confined to the contralateral STN, but is also present in the ipsilateral STN. While overall cumulative synchronization does not differ between the two hemispheres, their temporal dynamics diverge. Specifically, ipsilateral ERS lacks consistent time-locking across high-frequency sub-bands, exhibits a distinct ERS-ERD relationship and shows weaker, more variable coupling to muscle activity. These differences in coordination and motor specificity may be physiologically meaningful, to reduce interference and pathological co-activation between hemispheres. At the same time, it may represent a non-specific echo of contralateral motor commands, to increase neuronal preparatory readiness and leverage upcoming movements of its corresponding site.^55^

### Spatial dynamic of synchronization in basal ganglia

In the cortex of both healthy humans and non-human primates, it was shown that oscillatory activity acts not only through amplitude variations in the time domain, but also unfolds spatially across networks.^56-58^ Such spatial dynamics contribute to the fine-tuning of motor control through flexible allocation of neuronal resources.^33,34,59^ Whether the network architecture of the basal ganglia also exhibits similar behaviour-dependent spatial dynamics was unknown. Previously, we demonstrated that patients’ awake and resting state condition is characterized by a distinct spatial architecture, consisting of hotspots spanning low to high spectral components within the STN.^37,41^ Furthermore, we showed that this spectral architecture may change according to the state of consciousness, suggesting underlying dynamical capabilities of STN networks.^38^ Extending this view, we now present evidence that spatial distribution of spectral hotspots also adapts dynamically in space depending on the context of a specific motor activation behaviour.

We observed two key phenomena: in all participants, during the resting, pre-movement and movement state, the location of hotspots within the STN exhibit a certain volatility around the mean. Interestingly, this volatility diminishes during the transition from rest towards active motion, likely reflecting the role of the STN in facilitating motor execution by reducing neural variability.^60^ In parallel to the change in volatility, we observed a second phenomenon, which is the spatial propagation of each mean hotspot position primarily along the superior-inferior trajectory. Propagations are already clearly present at the transition from the resting state to the pre-movement state in the superior direction, probably in anticipation of a motor programme execution that is about to be initiated. During motor execution, both HG and FG hotspots further move towards superior, whereas SHFO hotspots moves towards the inferior direction. While both HG and FG hotspots transiently occupy the associative subregion of the STN, both SHFO and FHFO remain largely confined to the motor STN. These spatially and spectrally distinct trajectories likely reflect differentiated functional roles of oscillatory sub-bands, suggesting that local dynamics flexibly adapt to behavioural demands through recruitment of spatially organized networks within and across functional domains.^61,62^

Taken together, the band-specific topography at rest and the dynamical modulation during movement likely arise from segregated yet converging functional and somatotopic cortical-basal ganglia pathways.^61,63-65^ The HG hotspot may reflect gamma-dominant inputs from motor cortical regions projecting to the STN, whereas faster rhythms such as FG and HFO appear to originate locally within the STN, consistent with their limited cortical-subcortical connectivity.^31,66^ In particular, HFO may reflect synchronized firing of local neuronal ensembles, as well as phase and frequency shifts of oscillatory activity.^1,30,67,68^

Importantly, the amount of spatial propagation within the STN is not strictly time-locked to the temporal magnitude dynamic of the ERS. This suggests that the spatial propagation of hotspots reflects an additional functional degree of freedom in the neural encoding of movement, and thus a principled and physiologic *modus operandi* to flexibly adapt to changing contexts.^69,70^ Although the present study highlights spatial basal dynamics in the limited context of a simple motor task, it is plausible that they also scale together with more granular kinematic parameters of continuous behaviour, as recently similarly demonstrated for time domain features.^71^

### Spatial synchronization dynamics index motor dysfunction

We hypothesized that abnormal spatial encoding of synchronization hotspots may also be linked to motor dysfunction in PD. Specifically, we found that the extent of spatial propagation of sub-bands above 110 Hz is related to a reduced motor state improvement following levodopa intake. This suggests that exaggerated spatial propagation in the context of PD represents a dysfunctional network recruitment, probably as a compensatory reaction following the neurodegenerative process. Among all different sub-bands, spatial propagation of FG emerged as the most sensitive predictor for the clinical state. Compared with other sub-bands, synchronization in FG was proportionally characterized by the largest spatial propagation, and exhibited the strongest correlative strength between spatial propagation and muscle activity. It remains to be determined if the dominant clinical phenotype of PD would influence these spatial dynamics. However, we note that excessive levels of spatial network activation, which reflects a lack of spatial selectivity, is actually not exclusive to PD. Instead, links can be established to other movement disorders such as dystonia, which is clinically characterized by pathological co-activation of muscle agonists and antagonists, overflow movements and enlargement in the receptive field in sensory testing. In PD, we can also assume dependency and reversibility of excessive network activation and hypermetabolism according to the dopaminergic tone, since both at the levels of large networks and of neuronal firing, neuronal selectivity was shown to improve in the ON dopaminergic state.^72-75^ During DBS, movement-related synchronization tends to become even more pronounced.^76^ But whether stimulation would similarly reinstate physiological spatial dynamics, beyond alleviating motor symptoms, remains uncertain and warrants further investigations.^77^ Nevertheless, based on observations in this work, we anticipate translatable aspects of spectro-spatial metrics into biomarker libraries for clinical applications. In the evolving era of brain-sense guided neuromodulation, spatial network adjustments hold the potential to not only inform about physiological and clinical states in chronically implanted patients, but also explain signal-to-noise ratio variations of spectral biomarkers due to transient positional shifts. Hence, in addition to local temporal power variation, spatial dynamics may become an integrative feature of future adaptive algorithms.^78^

### Limitations

This study focuses solely on the synchronization dynamics within the STN network and the absence of simultaneous cortical recordings limits direct assessment of cortico-subthalamic interactions, which therefore remain hypothetical. It’s important to note that the motion tracking of hand closing/opening is primarily based on the lower arm flexor muscle EMG and not controlled for other spontaneous movement activity. The intraoperative recording set-up during DBS surgery introduces time constraints and potential variability in the assessments due to patients' fatigue as well as micro-lesion effects. Nevertheless, this set-up offers the key advantage of obtaining high-sampled monopolar signals for high-resolution spatiotemporal analyses, which currently are not achievable with chronically implanted BrainSense systems.

## Conclusions and outlook

Our findings demonstrate that movement-related neuronal synchronization within the STN involves multiple high-frequency bands with each reflecting some degrees of distinct dynamical properties. We evidence that synchronization also evolves in space within the local STN network and expand the view of a cortical-subcortical mechanism of information processing. Finally, we show that neurodegeneration may amplify spatial propagation which may serve as a potential new neurophysiological hallmark of motor impairment in PD. These insights into the spatial dynamics of basal ganglia oscillations opens up new avenues of spectro-behavioural investigations and clinical translations in motor and neuropsychiatric disorders.

## Supplementary Material

awag019_Supplementary_Data

## Data Availability

The data that support the findings of this study are available from the corresponding author, upon reasonable request.
