# Supplementary material for: Spatial propagation of movement-related basal ganglia activity predicts parkinsonian motor state
Source: Brain. 2026 Jan 20;149(5):1522–36. doi: 10.1093/brain/awag019 (PMC13140544; doi:10.1093/brain/awag019)
Supplement: awag019_Supplementary_Data [file awag019_supplementary_data.pdf]

Supplementary material

Spatial propagation of movement-related synchronisation in the subthalamic nucleus predicts parkinsonian motor states

Supplementary Table 1: Demographic data

Patient Number	Sex	Recording Site	Age	Time To Surgery (y)	Dominant Phenotype	MDS-UPDRS-III ON	MDS-UPDRS-III OFF	LEDD
1	m	l / r	61	9	Mixed	6	30	840
2	m	l / r	72	9	Tremor-Dominant	14	55	1600
3	f	l	73	11	Akinetic-Rigid	8	34	1530
4	m	l / r	64	16	Akinetic-Rigid	23	56	1700
5	m	l / r	54	11	Akinetic-Rigid	27	58	2520
6	f	r	70	9	Tremor-Dominant	6	30	470
7	m	l	34	7	Akinetic-Rigid	10	29	1410
8	m	l / r	55	2	Akinetic-Rigid	25	40	350
9	f	l / r	64	13	Akinetic-Rigid	7	40	480
10	m	l	53	6	Akinetic-Rigid	13	30	1200
11	f	r	59	8	Akinetic-Rigid	16	41	550
12	f	r	69	16	Akinetic-Rigid	3	25	1950
13	m	r	65	10	Akinetic-Rigid	14	38	2010
14	m	l / r	42	11	Akinetic-Rigid	11	31	1500
15	m	l	50	10	Akinetic-Rigid	17	49	1230
16	m	l	68	12	Mixed	27	59	1370
17	m	l / r	73	11	Akinetic-Rigid	8	32	1550
18	f	l / r	67	5	Tremor-Dominant	17	33	860
19	m	r	61	8	Akinetic-Rigid	18	63	1470
20	m	l / r	56	10	Akinetic-Rigid	10	42	850
21	f	l / r	75	4	Akinetic-Rigid	27	41	0
22	m	l / r	58	7	Akinetic-Rigid	4	26	1200
23	m	l / r	56	6	Akinetic-Rigid	16	27	1180

24	f	l / r	62	8	Akinetic-Rigid	27	60	1080
25	m	l	66	8	Akinetic-Rigid	24	49	1720
26	m	r	55	10	Akinetic-Rigid	25	53	980
27	m	l / r	55	12	Akinetic-Rigid	20	37	880
28	f	l / r	68	5	Akinetic-Rigid	13	32	300
29	m	r	56	11	Akinetic-Rigid	8	30	1700
30	f	l / r	71	14	Akinetic-Rigid	19	52	1330
31	f	r	69	24	Akinetic-Rigid	17	42	1540
32	f	l	50	4	Tremor-Dominant	8	40	950
33	m	l / r	57	9	Akinetic-Rigid	6	25	970
34	f	r	68	10	Akinetic-Rigid	4	26	950
35	f	l / r	46	4	Akinetic-Rigid	14	36	950
36	f	l / r	57	5	Akinetic-Rigid	28	35	120
37	f	l / r	75	20	Akinetic-Rigid	15	37	610
38	m	l / r	70	7	Tremor-Dominant	17	52	820
39	m	l	62	5	Tremor-Dominant	8	19	250
40	m	l / r	60	9	Akinetic-Rigid	5	43	400

UPDRS = Unified Parkinson's Disease Rating Scale;
LEDD = Levodopa equivalent daily dose (mg).

Supplementary Table 2: MNI coordinates (x, y, z) of hotspot positions for each ERS sub-band (HG, FG, SHFO, FHFO) across rest, pre-movement, and movement states.

		HG			FG			SHFO			FHFO		
		X	Y	Z	X	Y	Z	X	Y	Z	X	Y	Z
	**Rest**	12.43	-12.77	-7.67	12.39	-12.71	-7.67	12.35	-12.78	-7.73	12.31	-12.70	-7.66
	**Pre-Movement**	12.26	-12.67	-6.97	12.40	-12.70	-7.11	12.28	-12.86	-6.90	12.24	-12.68	-6.87
**Movement**	**-10**	12.40	-12.92	-6.94	12.58	-12.64	-6.73	12.22	-13.10	-7.50	12.06	-12.83	-7.50
	**-5**	12.26	-12.79	-7.05	12.51	-12.56	-6.44	12.31	-12.67	-7.55	12.20	-12.94	-6.80
	**0**	12.30	-12.85	-7.32	12.49	-12.73	-6.34	12.36	-12.84	-6.93	12.15	-12.87	-6.81
	**5**	12.32	-12.64	-6.72	12.49	-12.65	-6.33	12.34	-12.85	-7.14	12.01	-13.09	-6.87
	**10**	12.47	-12.58	-6.52	12.33	-12.81	-6.48	12.38	-13.11	-7.55	12.15	-13.09	-6.85
	**15**	12.47	-12.44	-6.58	12.41	-12.70	-6.62	12.36	-12.75	-7.42	12.14	-12.95	-6.80
	**20**	12.48	-12.79	-6.47	12.48	-12.48	-6.68	12.29	-12.87	-7.35	12.28	-13.06	-6.87
	**25**	12.39	-12.45	-6.04	12.43	-12.42	-7.06	12.50	-12.83	-7.03	12.21	-12.71	-6.76
	**30**	12.56	-12.63	-5.82	12.37	-12.55	-6.78	12.45	-12.56	-6.77	12.33	-12.85	-6.86
	**35**	12.47	-12.64	-6.90	12.49	-12.83	-6.96	12.51	-12.57	-6.48	12.32	-13.06	-6.72
	**40**	12.37	-12.77	-7.55	12.58	-12.61	-6.63	12.33	-12.66	-6.56	12.20	-12.94	-6.64
	**45**	12.30	-12.99	-6.91	12.75	-12.70	-6.61	12.26	-12.82	-7.02	12.31	-13.08	-7.15
	**50**	12.21	-12.90	-6.61	12.48	-12.86	-6.79	12.23	-12.80	-7.26	12.24	-12.74	-6.59
	**55**	12.16	-12.54	-6.40	12.43	-12.74	-6.76	12.19	-12.96	-6.88	12.36	-12.86	-6.57
	**60**	12.23	-12.60	-6.17	12.30	-12.84	-7.31	12.38	-12.86	-6.97	12.55	-12.75	-6.47
	**65**	12.37	-12.75	-6.45	12.54	-12.67	-6.63	12.50	-12.84	-6.87	12.31	-13.06	-6.90
	**70**	12.24	-12.62	-6.88	12.44	-12.65	-6.74	12.32	-13.02	-6.88	12.43	-12.97	-6.41
	**75**	12.35	-12.51	-7.25	12.19	-12.79	-7.21	12.08	-12.83	-6.14	12.50	-12.61	-6.31
	**80**	12.39	-12.76	-6.39	12.35	-12.78	-6.27	12.24	-12.78	-6.72	12.32	-12.81	-6.85
	**85**	12.51	-12.63	-6.24	12.34	-12.96	-7.55	12.25	-12.71	-7.15	12.44	-12.64	-6.96
	**90**	12.39	-12.43	-6.51	12.37	-13.00	-7.55	12.28	-12.94	-7.28	12.39	-12.82	-6.97
	**95**	12.12	-12.43	-6.81	12.31	-12.96	-7.42	12.38	-12.87	-7.18	12.38	-12.90	-6.75
	**100**	12.40	-12.61	-6.45	12.28	-12.68	-6.87	12.26	-13.00	-7.10	12.46	-12.87	-7.32
	**105**	12.48	-12.53	-6.76	12.36	-12.73	-7.09	12.24	-13.04	-6.73	12.38	-12.51	-6.29
	**110**	12.40	-12.59	-7.00	12.15	-12.51	-6.90	12.27	-12.90	-6.78	12.15	-12.77	-6.46

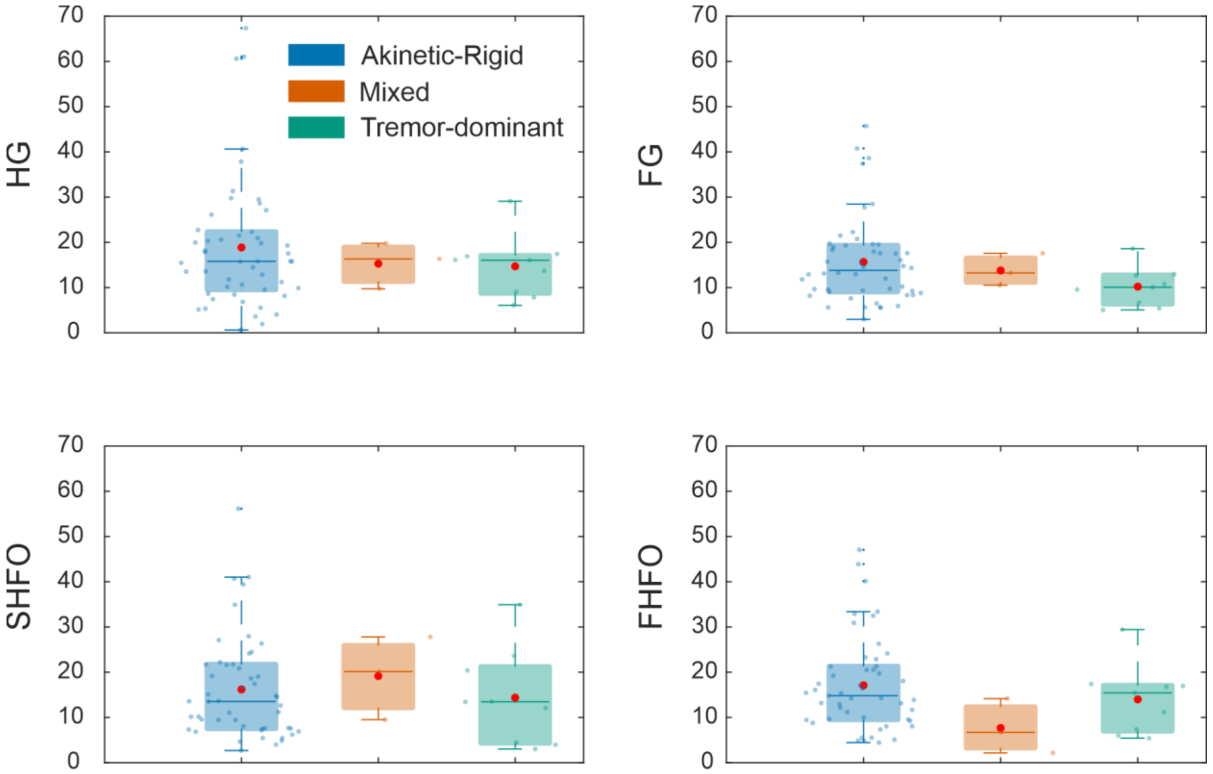

Supplementary figure 1: Movement-related spectral power (AUC) across frequency bands and clinical phenotypes. Grouped boxplots showing the cumulative event-related-synchronization measured as AUC for each spectral sub-band (HG, FG, SHFO, FHFO) across clinical phenotypes (Akinetic-rigid, Tremor-dominant, Mixed). Individual data points are overlaid as dots within each boxplot. No significant differences in movement-related AUC were observed between phenotypes for any of the frequency bands, indicating comparable spectral activation across clinical subgroups.

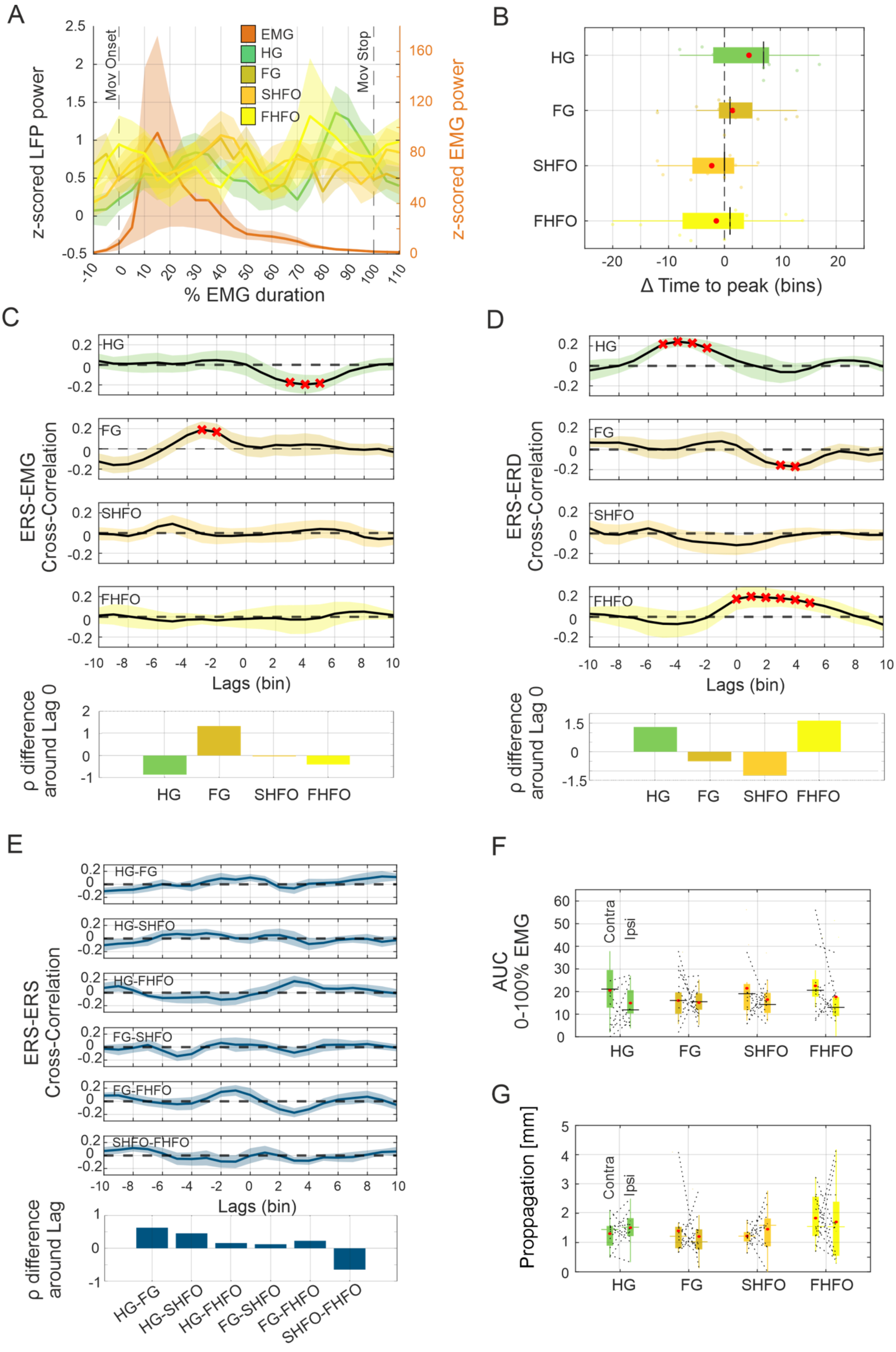
A
z-scored LFP power
z-scored EMG power
% EMG duration
Mov Onset
Mov Stop
EMG
HG
FG
SHFO
FHFO
B
HG
FG
SHFO
FHFO
Δ Time to peak (bins)
C
ERS-EMG
Cross-Correlation
HG
FG
SHFO
FHFO
Lags (bin)
ρ difference
around Lag 0
D
ERS-ERD
Cross-Correlation
HG
FG
SHFO
FHFO
Lags (bin)
ρ difference
around Lag 0
E
ERS-ERS
Cross-Correlation
HG-FG
HG-SHFO
HG-FHFO
FG-SHFO
FG-FHFO
SHFO-FHFO
Lags (bin)
ρ difference
around Lag
F
AUC
0-100% EMG
Contra
Ipsi
HG
FG
SHFO
FHFO
G
Proppagation [mm]
Contra
Ipsi
HG
FG
SHFO
FHFO

Supplementary Figure 2: Magnitude and Temporal Dynamics of Ipsilateral Movement-related Synchronization. (A) Ipsilateral to movement oscillatory spectral power changes of sub-bands—HG, FG, SHFO, and FHFO — aligned to movement onset (0% of the movement phase). (B) Time lag between the peak of EMG activity and the peak of ERS across hemispheres. No significant delay between spectral peaks and EMG peak. Total ERS, measured as area under the curve (AUC) (0 to 100% of movement) for each sub-band and across hemispheres. HG showed significantly greater synchronization compared to FG. (C) Cross-correlation between band-specific ERS and EMG activity (top) and mean standardized difference ± time bins around lag 0 (bottom). Significant coupling, yet with a different temporal extent, was found for HG and FG, with HG preceding muscle activity, while FG followed EMG activation. (D) Cross-correlation between band-specific ERS and ERD activity (top) and mean standardized difference ± time bins around lag 0 (bottom). No significant coupling was found in any of the sub-bands. (E) Cross-correlation of sub-band time courses (top) and mean standardized difference ±3 bins around lag 0 (bottom). Comparable temporal dynamics were observed across sub-bands. Shaded areas represent ± standard error of the mean (s.e.m.). (F) Total ipsilateral vs contralateral ERS, measured as area under the curve (AUC) (0 to 100% of movement) for each sub-band and across hemispheres. No significant differences were found across sub-bands. (G) Mean hotspot propagation during movement state, ipsilateral vs. contralateral. No significant differences were found across sub-bands. HG, high-gamma; FG, fast-gamma; SHFO, slow high-frequency oscillations; FHFO, fast high-frequency oscillations; EMG, electromyography.

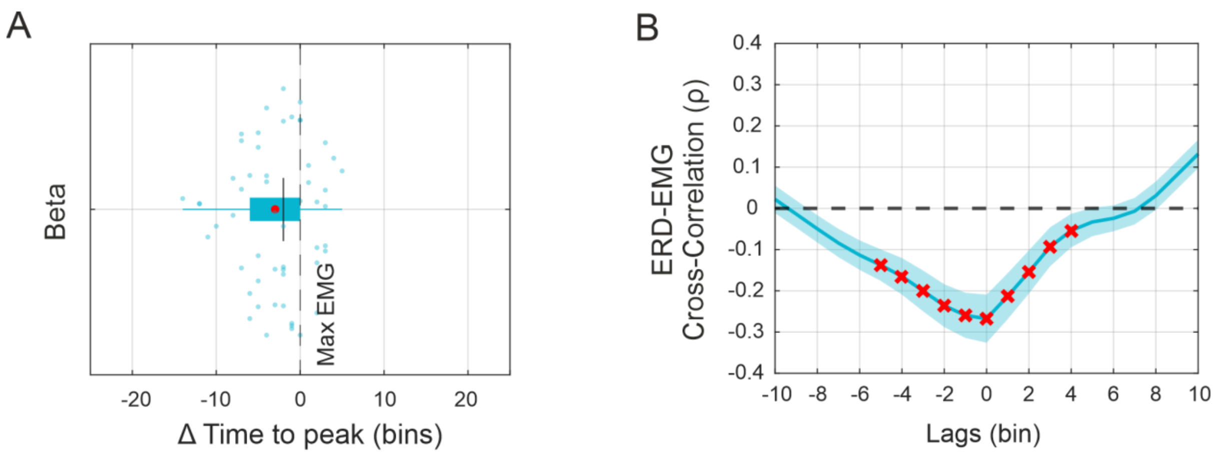

Supplementary Figure 3: Characteristics of Movement-related beta desynchronization. (A) Comparison of timing between peak in beta ERD and peak EMG. Beta ERD peaks significantly earlier than EMG. Red dot and black line represent the mean and median of distributions respectively. (B) Cross-correlation between beta ERD and EMG activity. Significant coupling, was found with peak correlations centred around 0 lag. Shaded areas indicate ± s.e.m. ERD, event-related desynchronization; EMG, electromyography.

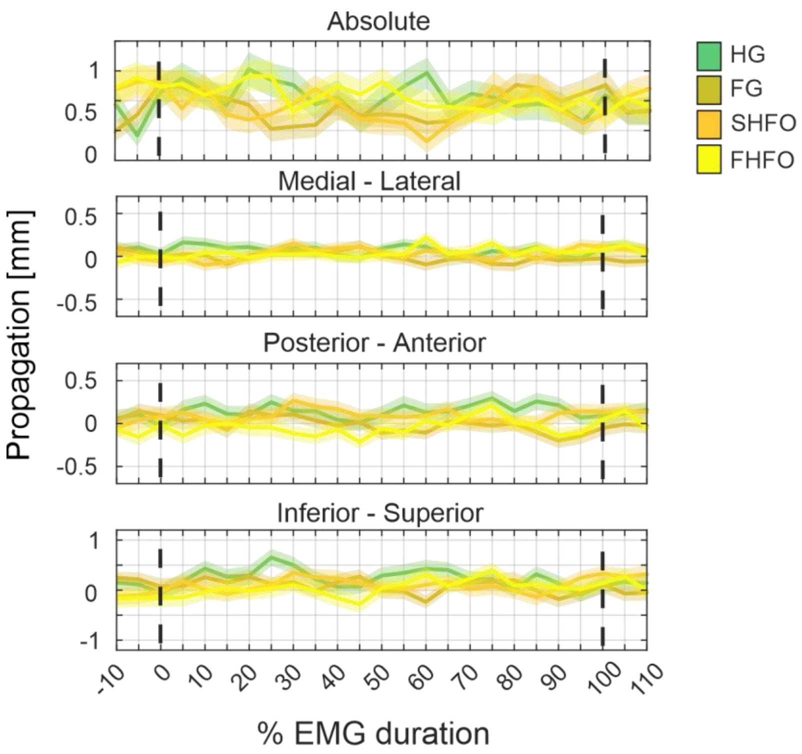

Supplementary Figure 4: Individual-level Hot Spot Propagation. Plots showing the temporal evolution of hotspot propagation by sub-band across the medial–lateral, anterior–posterior, and inferior–superior axes, as well as the absolute Euclidean distance. The temporal dynamics of spatial propagation at the individual level closely mirrored those observed at the group level across all sub-bands. Shaded areas indicate ± s.e.m. HG, high-gamma; FG, fast-gamma; SHFO, slow high-frequency oscillations; FHFO, fast high-frequency oscillations; EMG, electromyography.

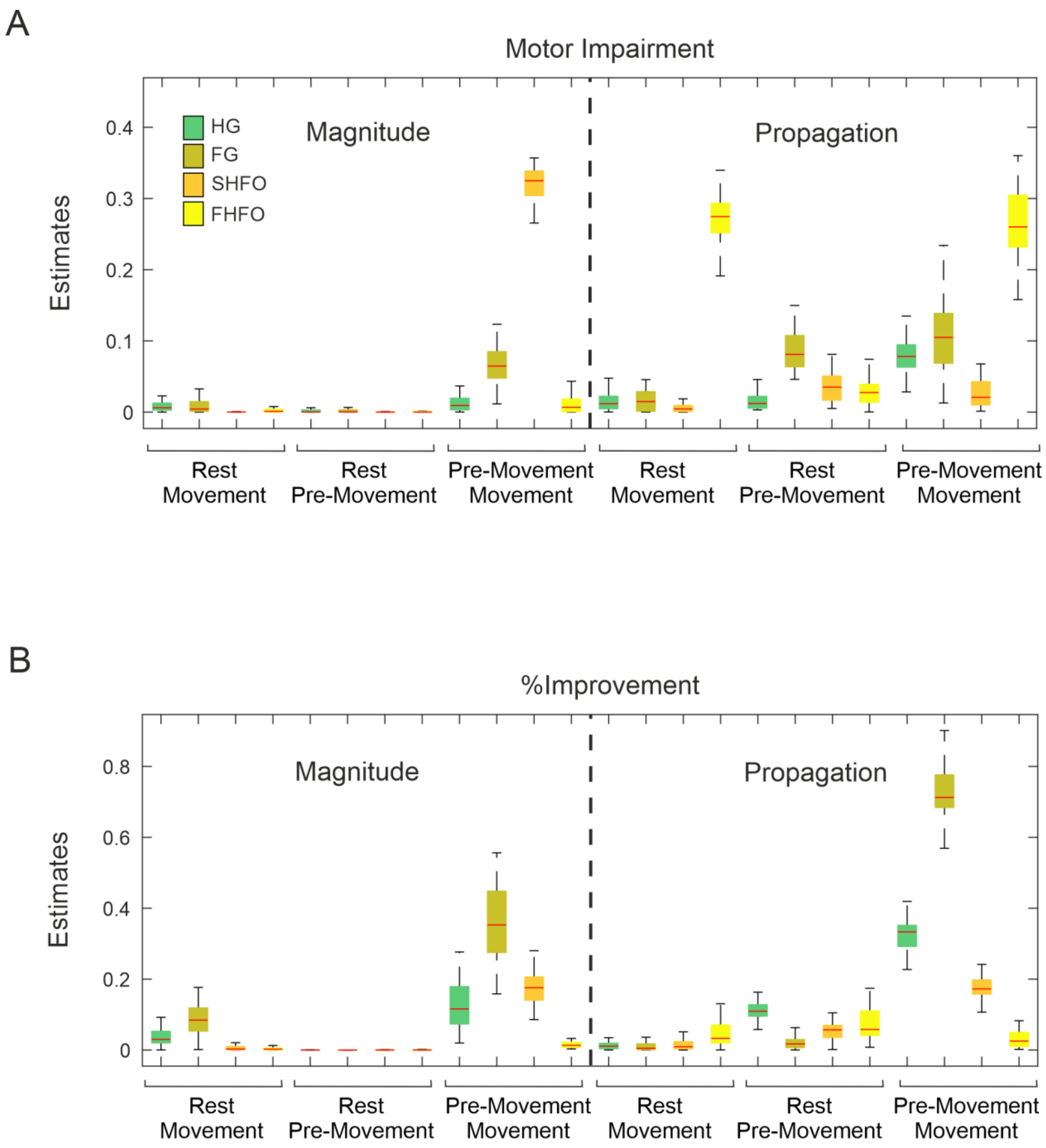

Supplementary Figure 5: Predictive Weights of Clinical Outcomes Using ERS Metrics. (A) Boxplots showing the distribution of predictive weights (estimates) for each feature used to predict motor impairment in the OFF-medication state (based on UPDRS scores), derived from leave-one-out cross-validation. (B) Boxplots showing the predictive weights for each feature used to predict motor % improvement following medication. Features include the magnitude and propagation of event-related synchronization (ERS) in four frequency sub-bands—HG, FG, SHFO, and FHFO—computed across three state transitions: Rest to Pre-Movement, Rest to Movement, and Pre-Movement to Movement. The weights represent the relative importance

of each feature in contributing to the predictive model. HG: high-gamma; FG: fast-gamma; SHFO: slow high-frequency oscillations; FHFO: fast high-frequency oscillations.
